# Ventriculitis: Infection or Inflammation

**DOI:** 10.3390/antibiotics10101246

**Published:** 2021-10-14

**Authors:** Mahesh Ramanan, Andrew Shorr, Jeffrey Lipman

**Affiliations:** 1 Intensive Care Unit, Caboolture Hospital, Caboolture, QLD 4510, Australia; 2 Adult Intensive Care Services, The Prince Charles Hospital, Chermside, QLD 4032, Australia; 3 School of Medicine, The University of Queensland, Brisbane, QLD 4072, Australia; j.lipman@uq.edu.au; 4 Critical Care Division, The George Institute for Global Health, University of New South Wales, Newtown, NSW 1466, Australia; 5 Washington Hospital Center, Medical Intensive Care Unit, Washington, DC 20010, USA; andrew.shorr@gmail.com; 6 Jamieson Trauma Institute and Intensive Care Services, Royal Brisbane and Women’s Hospital, Herston, QLD 4029, Australia; 7 Nimes University Hospital, University of Montpellier, 30029 Nimes, France

**Keywords:** ventriculitis, cerebrospinal fluid, ventriculostomy, catheter-related infection, antibiotics, nosocomial infection, neurosurgery

## Abstract

Ventriculitis, or infection of the cerebrospinal fluid, in the presence of external ventricular drains (EVD), is an important complication and associated with substantial mortality, morbidity, and healthcare costs. Further, the conditions that require the insertion of an EVD, such as neurotrauma and subarachnoid hemorrhage, are themselves associated with inflammation of the cerebrospinal fluid. Phenotypically, patients with inflammation of the cerebrospinal fluid can present with very similar symptoms, signs, and laboratory findings to those with infection. This review examines various controversies relating to the definitions, diagnosis, challenges of differentiating infection from inflammation, prevention, and treatment of ventriculitis in patients with EVDs.

## 1. Introduction

Cerebrospinal fluid (CSF) infections in the setting of neurosurgery and intracranial devices, such as external ventricular drains (EVD), intracranial pressure monitors, and CSF shunts, are an important and common complication [1] with significant associated mortality and morbidity [2]. These CSF infections are referred to as healthcare-associated ventriculitis and meningitis (referred to as “ventriculitis” in this review), as opposed to community-acquired meningitis, which refers to CSF infection in the absence of intracranial devices and neurosurgery [3].

EVDs are commonly used devices for neurocritical care patients with brain injury from a variety of sources, including subarachnoid hemorrhage, neurotrauma, intracerebral hemorrhage, ischemic stroke, central nervous system (CNS) infections, and hydrocephalus. The presence of intracranial devices such as EVDs can trigger an inflammatory response that can present with very similar or identical clinical and biochemical findings to ventriculitis [4]. Various definitions have been proposed for the diagnosis of ventriculitis, which include a range of clinical, biochemical, and microbiological criteria (Table 1). The breadth of the definitions available is reflective of the uncertainties faced by the clinician at the bedside who is tasked with differentiating an infectious ventriculitis from an inflammatory ventriculitis.

This review will discuss the challenges and controversies in differentiating true infectious ventriculitis from inflammation in patients with EVDs, and the implications on clinical practice for neurologists, neurosurgeons, critical care, and infectious disease physicians.

We searched PubMed with various combinations of search terms, including “ventriculitis”, “healthcare-associated meningitis”, “ventriculostomy infection”, “external ventricular drain”, “diagnosis”, and “incidence”. Relevant articles were screened by one author (MR) and assessed for relevance. Reference lists of these articles and other major publications in the field known to the authors were searched for additional relevant articles.

## 2. Ventriculitis

### 2.1. Definitions

The Center for Disease Control [5] defines ventriculitis based on the presence of either microbiological confirmation of pathological organism cultured from CSF or on the combination of several clinical, CSF biochemical, and serological criteria. However, as described in Table 1, a vast number of definitions have been described in the literature. These range from highly restrictive definitions that require microbiological evidence to liberal definitions that only require general features of inflammation [5,6,7,8]. This has led to substantial variability in the reported incidence of ventriculitis, and as such, incidence rates of ventriculitis must always be interpreted in relation to the definitions used.

### 2.2. Incidence

A meta-analysis from 2015 [10] demonstrated that the incidence of ventriculitis from the published literature was 11.4 cases per 1000 catheter days for patients with EVDs. However, this ranged from 8.8 to 17.0 cases per 1000 catheter days based on the definitions used. An incidence of 9.8 per 1000 catheter days was observed in a large prospective cohort study of EVDs conducted in the United Kingdom [9]. This study used a liberal, pragmatic definition for ventriculitis that required either a positive CSF culture or clinical suspicion in the presence of a variety of clinical and CSF and serum biochemical abnormalities. A more restrictive definition (requiring positive CSF culture in the presence of fever and CSF abnormalities) used in a prospective cohort study conducted in Italy demonstrated a rate of 7.3 per 1000 device-days (this study included both EVDs and lumbar catheters) [8]. A large German registry study [11] with >200,000 catheter days of observation reported a lower ventriculitis rate of 3.96 cases per 1000 catheter days using the CDC definition of ventriculitis.

Given the large disparities in defining ventriculitis, further studies are required to prospectively evaluate the commonly used definitions with respect to short- and long-term outcomes such as mortality and neurological recovery. A consensus definition that can rapidly and prospectively categorize patients according to risk of death and neurological dysfunction would have immense clinical utility for clinicians looking after patients with EVDs.

### 2.3. Why Does Ventriculitis Matter?

Ventriculitis is associated with high rates of mortality and morbidity [1,12]. A recent, large case series demonstrated a mortality rate of 30% among patients with ventriculitis, though that cohort included patients without EVDs as well [2]. It is important to recognize that ventriculitis occurs in patients who are at elevated risk of mortality due to their underlying neurocritical condition (such as subarachnoid hemorrhage, traumatic brain injury); therefore, mortality in these patients is attributable to many reasons, one of which is ventriculitis.

Ventriculitis complicating EVD insertion is associated with significantly longer hospital length of stay and cost of healthcare [13]. The risk of neurological impairment among survivors has increased five-fold [13], with Luque-Paz et al. [2] reporting persistent, long-term neurological impairment in 62% of patients. Deficits included cognitive disorder, seizures, gait disturbance, fatigue, and memory loss. The overall societal costs (including healthcare and additional care requirements for daily living) of such impairment have not formally been costed but are likely to be substantial.

### 2.4. Risk Factors

A large variety of risk factors for the development of ventriculitis have been described in the literature. Neurosurgery and the presence of an EVD itself are associated with an increased risk of ventriculitis [14,15,16,17,18]. Broadly, the risk factors of ventriculitis in patients with EVD can be divided into patient-, catheter-, maintenance-, and institutional-associated risk factors.

A recent study conducted in a French neurosurgical intensive care unit (ICU) shed some light on pathophysiological mechanisms that lead to ventriculitis. By performing systematic daily swabs of skin (at EVD insertion site), EVD stopcock, and CSF cultures, Mounier et al. [19] demonstrated that ventriculitis occurs mainly due to “extra-luminal progression of pathogens initially colonizing the skin site where CSF leaks”. CSF leak has previously been described as a strong risk factor for ventriculitis [13].

Age, sex, and diabetes, all known to be associated with the risk of developing nosocomial infections, may not be associated with the development of ventriculitis [20,21]. However, patients with an immunocompromised state and those having redo surgery are at increased risk [22].

Maintenance-related risk factors for ventriculitis include frequency of manipulation (including for flushing of EVD), presence of intraventricular blood, and possibly disconnection and leakage from the drainage system [1,23]. There is a bimodal distribution of time at which ventriculitis is diagnosed with a peak at 9–11 days followed by a later peak beyond 14 days [1,17,23,24,25,26].

Catheter-related factors that may be associated with the risk of ventriculitis include catheter impregnation (described in Section 4.1) and routine EVD exchange. Some institutions have protocolized EVD exchange (typically at or close to 7 days) policies, but these have not been shown to reduce the incidence of ventriculitis [1,27,28].

## 3. Infection or Inflammation?

### 3.1. Central Nervous System Inflammation

Fever, one of the cardinal signs of infection, occurs in up to 87% of patients with severe brain injury, the cause of which may be ischemic stroke, neurotrauma, subarachnoid hemorrhage, or post neurosurgery [29]. In some cohorts, around half of brain-injured patients with fever have a non-infectious fever [30]. Fever is an important risk factor for worse outcomes in conditions such as stroke [31], and temperature control, both pharmacological and physical measures, is frequently applied [32].

The immune response to brain injury is characterized by the release of proinflammatory cytokines such as tumor necrosis factor alpha and interferon gamma, intercellular adhesion molecules, and chemokines [33,34,35]. Systemic features such as fever, tachycardia, tachypnoea, rise in peripheral blood leukocyte count, fall in lymphocyte count, and acute phase reactions (for example, rise in C-reactive protein, procalcitonin) are commonly observed in brain injury [36,37]. Some inflammation may be necessary to remove debris arising from the traumatic event from the central nervous system (CNS); however, persistent inflammation can be maladaptive and lead to harm through immunosuppression and increased risk of infections, both locally within the CNS and also distally [38,39,40].

### 3.2. CSF Features of Inflammation

In addition to the clinical phenotype of inflammation and an acute phase reaction in the blood, an inflammatory reaction can also be detected in the CSF of patients with brain injury. This is characterized by CSF pleocytosis (typically an early preponderance of polymorphonuclear cells), elevated protein concentration, and low glucose concentration. These features are identical to those observed in ventriculitis [41]. Gram stain and bacterial cultures are negative in states of inflammation but can also be negative in ventriculitis due to prophylactic antibiotics or antibiotic-impregnated EVDs [42]. Additional indices such as the cell index, a ratio calculated as (CSF white cell to red cell count)/(peripheral blood white cell to red cell count) may be useful in distinguishing inflammation from infection [43,44].

Neuroimaging, including magnetic resonance imaging (MRI), may have a role in detecting inflammation and infection in the CNS [45], especially when CSF and other biomarkers are inconclusive. However, performing MRI on neurocritical care patients can be logistically challenging and highly resource-intensive. It is typically not used as a routine investigation but may be considered in select cases.

### 3.3. CSF Features of Infection

Thus, clinicians are faced with a profound dilemma whereby the clinical phenotype of inflammation systemically and in CSF is very frequently present in neurocritical care patients with an EVD. However, these patients are also at high risk of developing ventriculitis, an infection that has the same systemic and CSF features as inflammation but also has significant consequences, i.e., neurological sequelae and increased mortality. Studies have shown that clinician recognition of infectious diagnoses in critical care environments, where inflammation is commonly present, is highly variable [46,47]. Even when evaluating sepsis and septic shock, where consensus guidelines have existed for many years, Rhee et al. [47] concluded that the diagnosis of sepsis was subjective and variable between clinicians. However, the consequences of ventriculitis are arguably of greater consequence to patients, and therefore, there is a strong clinical imperative to identify and treat ventriculitis. Yet the antibiotic components of that treatment are associated with substantial harm, particularly in critical care, including selection of organisms with antimicrobial resistance, secondary infections such as Clostridium difficile colitis, alteration of the microbiome, and risk of allergic reactions and other adverse events [48,49].

These challenges in differentiating inflammation from infection in the CSF have led to investigations of biomarkers and other novel technologies. While the gold standard remains the identification of pathogenic bacteria on gram staining and bacterial cultures, relying solely on microbiological diagnosis will result in missed cases of ventriculitis and delayed therapy with possible negative consequences for patients.

One simple biomarker for detecting ventriculitis may be CSF lactate, which has been reported to have both sensitivity and specificity of 86% [50] when a cut-off of CSF lactate >4.15 mmol/L was used. A meta-analysis of community-acquired meningitis patients reported high sensitivity (93%) and specificity (99%) for CSF lactate (cut-off >3.8 mmol/L) in distinguishing aseptic from bacterial meningitis [51]. However, CSF lactate is most likely to be useful in patients with very high or low values, with lesser utility in the 3–6 mmol/L range [52].

A variety of cytokines have been demonstrated in the CSF of patients with bacterial meningitis, including interleukin-6 (IL6), interleukin-8 (IL8), interleukin-1-beta (IL1b), and tumor necrosis factor alpha (TNFa) [53,54,55]. CSF IL1b, particularly, seems to be associated with high specificity and sensitivity for distinguishing bacterial from aseptic meningitis. CSF IL6 is a promising biomarker with a small study showing 100% sensitivity and specificity for the diagnosis of ventriculitis in patients with aneurysmal subarachnoid hemorrhage [56]. However, none of these cytokines have been specifically evaluated for the diagnosis of ventriculitis in patients with EVDs. Currently, their use should be considered exploratory, and high-quality prospective studies are required. A further limitation is the cost and availability of measuring CSF cytokine concentrations.

Investigations based on metabolomics may have a role in distinguishing neuroinflammation from infection [57]. Of particular interest are lipid-based biomarkers, such as free phosphatidyl choline levels [58,59]. The CNS contains high concentrations of lipids, including phosphatidyl choline, ceramides, and sphingomyelins, all of which have important roles in the inflammatory response. Dysregulation of these lipids has been shown to occur in various CNS diseases. Phosphatidyl choline levels have been shown to be elevated in meningitis, particularly bacterial meningitis [59]. While currently not routinely available for clinical use, CSF phosphatidyl choline assays herald significant promise for accurate diagnosis of CSF infection.

Rapid identification of bacteria in biological samples can be performed using matrix-assisted laser desorption/ionization time-of-flight mass spectrometry (MALDI-TOF) [60]. While detection of pathogenic bacteria (i.e., those not associated with contaminated samples) in blood always signifies infection, in other tissues, such detection may also occur in states of colonization also. This technology is still in its infancy for clinical use in CSF infections. A small single-center study of patients with bacterial meningitis showed that MALDI-TOF was able to detect 17 out of 21 Gram-negative bacilli but only 1 out of 11 Gram-positive cocci implicated in the infections [61]. MALDI-TOF appears to have better diagnostic performance for enteroviral meningitis with an overall diagnostic accuracy of 93% [62]. MALDI-TOF has not been formally evaluated in patients with EVDs and suspected ventriculitis. This may be one avenue of future research that could lead to rapid and accurate diagnosis of ventriculitis and facilitate prompt targeted antimicrobial therapy.

Another emerging technology includes rapid pathogen detection using metagenomic next-generation sequencing of pathogen nucleic acids [63]. Still early in its development for clinical use, such technology may have a transformative role in the rapid detection of CSF infections in the future.

A significant limitation of some of these strategies is that they are often evaluated in medical patients with clinical diagnoses of meningitis and/or encephalitis. Whether findings from this cohort are directly applicable to neurocritical care patients with EVDs, who have substantial disruption to the blood-brain barrier at presentation, is debatable. Thus, further clinical studies performed in the neurocritical care unit to evaluate the validity of emerging techniques in this unique population are likely to be needed.

## 4. Prevention and Treatment

### 4.1. Catheter Type

There are three categories of EVDs available: plain, antibiotic-impregnated, and silver-impregnated. The impregnated EVD reduces the development and growth of bacterial colonies along the EVD and hence addresses the pathophysiological mechanism through which most cases of ventriculitis arise [64,65].

A meta-analysis has demonstrated that antibiotic-impregnated EVDs result in a significant decrease in positive CSF cultures [66]. However, whether there is a true reduction in ventriculitis or a reduction in culture positivity rate is a matter of debate [41]. Silver-impregnated catheters, while showing pre-clinical promise, have not been demonstrated to offer protection against ventriculitis in clinical studies [66,67]. A recent large, high-quality randomized trial of antibiotic- and silver-impregnated shunt catheters compared against standard catheters for patients receiving ventriculoperitoneal shunt insertion showed a significant reduction in infection rate over a median 22-month follow-up with antibiotic-impregnated catheters but not silver-impregnated catheter [68]. Based on available evidence from small RCTs and a meta-analysis in patients with EVD, and extrapolation from a large RCT in patients with ventriculoperitoneal shunts, and health economic benefits [65], it may be recommended that antibiotic-impregnated EVDs be used if available [3]. However, it must be acknowledged that the evidence base supporting this is not strong. The evidence base currently does not support the use of silver-impregnated EVDs outside of clinical trials.

### 4.2. Prevention Bundles

Recognizing that it may be difficult to perform adequately powered trials that demonstrate benefits from a single intervention, “bundles of care” that combine several different interventions and processes of care have been developed in various medical disciplines, such as the “ABCDEF” bundle used in critical care [69]. Bundles may be particularly attractive for preventing ventriculitis as it is a common and frequently measured complication that occurs in a group of well-defined (i.e., EVD present) but relatively uncommon conditions.

Numerous bundles that target the prevention of ventriculitis in patients with EVD have been defined [70,71,72,73]. They typically feature protocolization of various aspects of care, including choice of EVD (e.g., antibiotic-impregnated), prophylactic and periprocedural antibiotics, skin preparation, aseptic and operative techniques, EVD tunneling distance, maintenance cares (e.g., sampling frequency, monitoring, disconnections), and infection surveillance. Benefits ranging from reduced rates of ventriculitis to shorter duration and fewer doses of antibiotics without an increase in ventriculitis and reduced rates of multiresistant organism infections have been reported from the use of bundles [70,71,72,73]. With the absence of harm and possibility of benefit, institutions that manage patients with EVDs should strongly consider the adoption of a protocolized bundle aimed at preventing ventriculitis. A large, well-designed, randomized trial to provide definitive evidence on EVD bundles is warranted.

### 4.3. Antibiotics

The blood-brain barrier, consisting of three layers including the cerebrovascular endothelium with tight junctions, the blood-CSF barrier at the choroid plexus epithelium, and the arachnoid epithelium, is a major consideration when administering antimicrobial therapy to patients with ventriculitis [74]. The blood-brain barrier maintains strict homeostasis in the CNS and prevents the entry of various toxins and pathogenic molecules into the CNS [75]. Many antibiotics, particularly hydrophilic antibiotics, fail to penetrate the blood-brain barrier in therapeutic concentrations under normal circumstances [76]. In situations of CSF inflammation, such as ventriculitis, penetration of certain antibiotics such as vancomycin is somewhat higher. Loading and maintenance doses of antibiotics for ventriculitis need to be higher than for infections at non-CNS sites. Where possible, therapeutic drug-level monitoring should be used [77] to ensure adequate concentrations of antibiotics are delivered to the CNS.

Antibiotic use for the prevention of ventriculitis varies strongly according to institution and clinician preferences. Periprocedural antibiotics, including a pre-procedure dose followed by 24–48 h of post-procedural antibiotics, have been shown to reduce infection rates [78,79,80]. Antibiotic choice is guided by local antimicrobiograms; however, a first-generation cephalosporin with Gram-positive activity such as cefazolin is often used. In areas with a high rate of methicillin-resistant *Staphylococcus aureus* colonization, a glycopeptide antibiotic such as vancomycin may be used. Prophylactic antibiotics beyond 24–48 h may result in higher rates of complications such as Clostridium difficile infection [79,80,81] [B98–104], Gram-negative infection [79], and infection with multiresistant organisms [82]. Prolonged prophylactic regimes are not associated with reductions in mortality or hospital length of stay [83]. For select patients with risk factors such as prolonged hospital stay prior to EVD insertion, anti-pseudomonal penicillin or cephalosporin prophylaxis should be used. Empirical treatment of suspected ventriculitis, while awaiting confirmation or antimicrobiogram, typically consists of an antipseudomonal beta-lactam and vancomycin [3].

Intraventricular antibiotics have been used in cases of ventriculitis resistant to conventional treatment (intravenous antibiotics and change of EVD). CSF penetration of antibiotics is highly variable due to the presence of the blood-brain barrier. Higher CSF levels of antibiotics can be achieved with intraventricular injection compared to intravenous dosing. Some antibiotics, such as aminoglycosides, cannot be used intravenously for ventriculitis due to very limited CSF penetration. However, they have been successfully used via the intraventricular route, as demonstrated in small case series and case reports [84,85]. Apart from higher CSF levels, intraventricular administration of vancomycin for Staphylococcal ventriculitis results in a faster reduction in CSF pleocytosis and CSF sterilization [86]. Intraventricular antibiotics are typically reserved for non-resolving cases of ventriculitis and are not used for prophylaxis.

## 5. Conclusions

Ventriculitis in the presence of an EVD in neurocritical care patients can be extremely challenging to diagnose as both inflammation and infection can present with very similar phenotypes in this population. While several tests may be helpful, none are sufficiently accurate for definitive diagnosis. Further research is required to facilitate rapid, accurate differentiation of ventriculitis from inflammation and strategies to prevent ventriculitis. Key messages are presented in Table 2.

## Figures and Tables

**Table 1:** Definitions of ventriculitis.

Study/Guidelines	Definition
Center for Diseases Control [5]	Must meet at least one of the following criteria: 1. Patient has organism(s) identified from cerebrospinal fluid (CSF) by a culture or non-culture-based microbiologic testing method, which is performed for purposes of clinical diagnosis or treatment, for example, not active surveillance culture/testing (ASC/AST). OR 2. Patient has at least two of the following: − fever (>38 °C) or headacheheadache; − meningeal sign(s) *; − cranial nerve sign(s) *. And at least one of the following: − increased white cells, elevated protein, and decreased glucose in CSF (per reporting laboratory’s reference range); − organism(s) seen on Gram stain of CSF; − organism(s) identified from blood by a culture or non-culture-based microbiologic testing method, which is performed for purposes of clinical diagnosis or treatment; − diagnostic single antibody titer (IgM) or four-fold increase in paired sera (IgG) for organism.
Gozal et al. [6]	Positive CSF culture in patient with EVD in situ and at least one of the following: temperature >38.6 °C CSF glucose either <50 mg/dL or <50% of serum glucose tested within 24 h of CSF glucose
Jamjoom et al. [7]	Positive CSF culture and/or gram stain OR Clinical suspicion of ventriculitis due to the presence of any of the following: − CSF pleocytosis; − elevated serum inflammatory markers; − fever; − meningism; − altered level of consciousness.
Citerio et al. [8]	Presence of all of the following: − positive CSF culture; and − CSF/blood glucose ratio less than 0.5; and − neutrophilic CSF pleocytosis (>5 cells/microlitre); AND − fever (body temperature > 38 °C).
Mounier et al. [9]	Positive CSF culture associated with CNS-targeted antibiotic treatment

* with no other recognized cause.

**Table 2:** Key Messages.

Key Messages
Multiple definitions for ventriculitis are in use broadly divided into those that require positive bacterial culture and those that do not
Ventriculitis incidence ranges from ~4 to 17 per 1000 catheter days depending on definition
Most cases of ventriculitis are seeded from site of EVD insertion along the tract
Ventriculitis is associated with long-term neurological dysfunction in up to 60% of survivors and significantly increased healthcare costs
Fever, neurological signs, tachycardia, tachypnoea, acute phase reactants, CSF pleocytosis, and reduced glucose concentration are common to infection and inflammation, thus differentiating ventriculitis from an inflammatory state is typically challenging
Apart from positive culture, cell index, CSF lactate, CSF cytokine levels, and rapid diagnostic tests such as MALDI-TOF may have a role in differentiating infection from inflammation, but more research is required
Preventative strategies such as antibiotic-impregnated catheters, EVD bundles, and periprocedural antibiotic prophylaxis should be employed
Antibiotic therapy for ventriculitis should take into account the blood-brain barrier and adequate dosing administered to achieve therapeutic CSF concentrations
